# An Efficient Approach for the Development of Locus Specific Primers in Bread Wheat (*Triticum aestivum* L.) and Its Application to Re-Sequencing of Genes Involved in Frost Tolerance

**DOI:** 10.1371/journal.pone.0142746

**Published:** 2015-11-13

**Authors:** Steve Babben, Dragan Perovic, Michael Koch, Frank Ordon

**Affiliations:** 1 Julius Kühn-Institut (JKI), Federal Research Centre for Cultivated Plants, Institute for Resistance Research and Stress Tolerance, Quedlinburg, Sachsen-Anhalt, Germany; 2 Deutsche Saatveredelung AG (DSV), Lippstadt, Nordrhein-Westfalen, Germany; Sabanci University, TURKEY

## Abstract

Recent declines in costs accelerated sequencing of many species with large genomes, including hexaploid wheat (*Triticum aestivum* L.). Although the draft sequence of bread wheat is known, it is still one of the major challenges to developlocus specific primers suitable to be used in marker assisted selection procedures, due to the high homology of the three genomes. In this study we describe an efficient approach for the development of locus specific primers comprising four steps, i.e. (i) identification of genomic and coding sequences (CDS) of candidate genes, (ii) intron- and exon-structure reconstruction, (iii) identification of wheat A, B and D sub-genome sequences and primer development based on sequence differences between the three sub-genomes, and (iv); testing of primers for functionality, correct size and localisation. This approach was applied to single, low and high copy genes involved in frost tolerance in wheat. In summary for 27 of these genes for which sequences were derived from *Triticum aestivum*, *Triticum monococcum* and *Hordeum vulgare*, a set of 119 primer pairs was developed and after testing on Nulli-tetrasomic (NT) lines, a set of 65 primer pairs (54.6%), corresponding to 19 candidate genes, turned out to be specific. Out of these a set of 35 fragments was selected for validation via Sanger's amplicon re-sequencing. All fragments, with the exception of one, could be assigned to the original reference sequence. The approach presented here showed a much higher specificity in primer development in comparison to techniques used so far in bread wheat and can be applied to other polyploid species with a known draft sequence.

## Introduction

### Genomic resources in wheat

Wheat (*Triticum aestivum* L.) is the cereal with the largest acreage worldwide [1]. It belongs to the family *Poaceae* and has a complex allohexaploid genome of about 17 Giga-base pairs (Gbp). The repeat content is approximately 80% which consists primarily of retroelements. The gene density is between 1 per 87 Kilo-base pairs (Kbp) and 1 per 184 Kbp [2, 3]. During evolution wheat became an alohexaploid organism (2n = 6x = 42) with the A, B and D genome. In brief, 300.000–500.000 years ago the first hybridisation between the wild diploid wheat (*Triticum urartu*, 2n = 2x = 14, genome A^u^A^u^) and an ancestor closest related to goat grass (*Aegilops speltoides*, 2n = 2x = 14, genome SS) took place [4, 5] leading to the generation of wild emmer wheat (*Triticum dicoccoides*, 2n = 4x = 28, genome A^u^A^u^BB) [6]. Tribal communities formerly making a living of gathering and hunting began to cultivate the wild emmer about 10,000 years ago. Human selection led to cultivated emmer (*Triticum dicoccum*). By a spontaneous hybridisation of cultivated emmer with another goat grass (*Aegilops tauschii* 2n = 2x = 14, genome DD) in combination with a natural mutation, bread wheat (*Triticum aestivum*, 2n = 6x = 42, genome AABBDD) was created [7]. Due to the hexaploid genome and a very high homology of the three sub-genomes in wheat, the genome sequence information has an inestimable value for molecular breeding, comparative genomics and association studies.

Nowadays, the National Center for Biotechnology Information (NCBI, http://www.ncbi.nlm.nih.gov/) database is a key virtual library of genomic, transcriptional and protein sequence data for more than 33,000 organisms [8]. NCBI serves as a web-platform for the identification of target gene sequences in organisms of interest, e.g. *Triticum aestivum*, *Triticum monococcum*, *Hordeum vulgare* etc. An additional wheat database is the CerealsDB web page created by members of the Functional Genomics Group at the University of Bristol (http://www.cerealsdb.uk.net), which includes online resources of genomic information, i.e. varietal SNPs, DArT markers, and EST sequences all linked to a draft genome sequence of the cultivar Chinese Spring [9]. Another web based portal is URGI, which includes datasets such as chromosome survey sequences, reference sequences, physical maps, genetic maps, polymorphisms, genetic resources, many phenotypic data and various genomic arrays (http://wheat-urgi.versailles.inra.fr). The chromosomal sequence information is granted by the International Wheat Genome Sequencing Consortium (IWGSC). All mentioned databases are suitable for the identification of homologous chromosome sequences in bread wheat. In addition to these resources, an important tool for wheat is the upcoming Genome Zipper of wheat (http://wheat-urgi.versailles.inra.fr). In the past few years, a lot of sequence information of wheatsorted chromosome arms [10–12], *T*. *urartu* [13] and *Ae*. *tauschii* [14] became available and was integrated in the above mentioned databases.

### Function and structure of frost tolerance genes

Low temperature is one of the most important limiting factors of wheat cultivation in North America and Eastern Europe. To ensure high yields in these areas, introduction of efficient frost tolerance alleles into elite cultivars is a prerequisite. Cold stress inhibits metabolic reactions and prevents wheat from fulfilling its genetic potential. To avoid yield losses, wheat needs acclimatisation to low temperatures, which prevents premature transition to the reproductive phase. This must happen before the threat of freezing stress during winter has passed [15]. Frost tolerance is a complex system involving many genes out of which six gene families/groups have been analysed in this study. According to their function, these genes belong to two separated metabolic pathways. The *Ppd* and *Vrn* genes are responsible for flowering, whereas the *Cbf*s, *Ice*s, *Tacr7*, *Dem*, *Cab* and *Dhn* genes are involved directly in frost tolerance. Regarding copy number, the analysed genes could be assigned as follows: *Dem* and *Tacr7* are single copy; *Ppd*, *Vrn* and *Ice* are low copy, while *Cbf*s, *Cab* and *Dhn* are high copy genes.

A high number of low temperature-induced genes was identified and characterized in plants [16, 17]. These are referred to as LATE EMBRYOGENESIS-ABUNDANT (*LEA*), Dehydrin (*Dhn*), Responsive to Abscisic Acid (*RAB*), Low Temperature–Responsive (*LT*) and Cold-Responsive (*COR*) genes. Several of the *COR* genes are dehydrins, which are a distinct biochemical group of LEA proteins [18–20] for which 54 different unigenes are described, of which 23 are involved in frost tolerance [21]. Dehydrins have either one but mostly two exons [22]. *Cab* genes or CAM-like (*CML*) genes, encoding proteins composed mostly of EF-hand Ca^2+^-binding motifs, may contain one to six exons [23]. *Cbf* genes are very important in the induction of *COR* genes through binding of C-repeat/dehydration-responsive elements (CRT/DRE) [15]. The complex *Cbf* gene family consists of 27 paralogs with 1–3 homologous copies per sub-genome. In total, the family contains at least 65 *Cbf* gene family members [24]. Knox et al. [25] detected that approximately half of the eleven *Cbf* orthologues at the FR-H2 locus in barley are duplicated. In addition, they reported that the variation in *Cbf* genes, which do not carry any introns, is widespread in the *Triticeae* [26]. This gene family is regulated by two wheat specific *Ice* genes under cold conditions [27, 28]. Both *Ice* genes have four exons [29, 30]. *Tacr7* belongs to the group of LT genes [31]. The *Dem* genes have an important role in the development of apical meristems and are thereby involved in the vegetative/reproductive transition of the shoot apex [27].

Flowering genes may be involved in frost the tolerance pathway because the flowering pathway contains vernalization and photoperiod response genes at crucial positions [32]. This pathway is regulated by five major *Vrn* genes (*Vrn1*, *Vrn2*, *Vrn3*, *Vrn4* and *Vrn5*) and two *Ppd* genes (*Ppd1* and *Ppd2*) [33]. The gene structure of the five vernalization genes varies from *Vrn1* having eight exons [34], via *Vrn3* with three exons [35] and *Vrn2* with two exons [36] to *Vrn4* and *Vrn5* of which the structure is unknown. The *Ppd1* gene shows eight exons [37], while the structure of *Ppd2* is unknown. The interaction between the flowering and the frost tolerance pathway is based on *Vrn1* and *Cbf* genes. The *Vrn1* gene may reduce transcript levels of *Cbf*s and *COR* genes under long day conditions.

### The draft wheat sequence and development of genomic markers

Nowadays, molecular markers, i.e. marker-assisted selection (MAS), are basic tools in plant breeding during germplasm characterization and cost efficient selection of important traits/genes. Furthermore, after gene isolation re-sequencing of specific fragments allows efficient allele mining [38]. However, the development of gene specific primers in wheat is hampered by the large genome size of 17 Gbp, the high repeat content of about 80% [2, 3], by the close homology of the three genomes (A, B and D) and by the high rate of similarity within genes and gene family members [10]. Comparative analysis of wheat sub-genomes shows high sequence homology and structural conservation and no significant differences in the rate of duplications between the sub-genomes are observed [11]. Recent efforts of the scientific community and the IWGS in sequencing of the 3 donor genomes as well as of the hexaploid wheat offer a solution in deciphering the intron-exon-structure of genes. By using differences of intron sequences among the homologous and paralogous copies of the various genes, it is possible to reconstruct the gene structure and identify differences between homologues. Continuous improvements of BLAST algorithms enhance the use of the above mentioned wheat genomic resources facilitating efficient primer development.Furthermore, specific primers are the basis for the development of molecular marker assays based on SNPsi.e. cleaved amplified polymorphic sequence (CAPS) [39], pyrosequencing [40] or competitive allele-specific polymerase chain reaction (KASP) [41], which are the base for marker assisted selection (MAS) procedures, anchoring physical and sequence contigs [12], germplasm characterization [42].

## Material and Methods

### Plant material and DNA extraction

In this study three cultivars (`Chinese Spring`, `Moskovskaya 39`and `VAKKA`) were used in initial testing of designed primer pairs, while a set of 24 genotypes, comprising two spring and 22 winter wheat cultivars, was used for re-sequencing of amplicons of frost tolerance genes (Table 1). For the physical assignment to chromosomes and chromosome segments 21 NT-lines [43] and 46 deletion-lines [44] were used (S1 Table) having the genetic background of ‘Chinese Spring’. The DNA was extracted at the three leaf stage according to Stein et al. [45].

**Table 1:** Plant material for PCR amplification and re-sequencing.

No.	Genotype	Country	Variety
1	Chinese Spring*		spring
2	Zentos	Germany	winter
3	Simila	Czech Republic	winter
4	Roughrider	USA	winter
5	Norstar	USA	winter
6	Moskovskaya 39*	Russia	winter
7	Bezenchukskaja 380	Russia	winter
8	Cheyenne	USA	winter
9	ÄRING II	Sweden	winter
10	VAKKA*	Finland	winter
11	Bezostaja 1	Russia	winter
12	Capelle Desprez	France	winter
13	Centurk	USA	winter
14	Mironovska 808	Ukraine	winter
15	Pobeda	Serbia	winter
16	Renesansa	Serbia	winter
17	Sava	Serbia	winter
18	Triple Dirk B (GK 775)	Australia	winter
19	Triple Dirk S	Australia	spring
20	ISENGRAIN	France	winter
21	APACHE	France	winter
22	SKAGEN	Germany	winter
23	JULIUS	Germany	winter
24	Biryuza	Russia	winter
25	Moskovskaya 40	Russia	winter

Complete set of 24 genotypes (without `Chinese Spring`) were used for sequencing.

* Genotypes for primer testing

### Sequence retrieval of genes involved in frost tolerance

As a starting point a set of 27 genes involved in frost tolerance was selected. 9 *Triticum aestivum* sequences together with 9 sequences from *Triticum monococcum* and 9 from *Hordeum vulgare*, known to be involved in frost tolerance from previous studies, served as a back bone for the identification of bread wheat frost tolerance candidate gene sequences (Table 2). If only the coding regions (mRNA-, EST- or protein-sequences) were available, the data bases of the International Wheat Genome Sequencing Consortium (IWGSC, http://www.wheatgenome.org/) and/or the Bristol Wheat Genomics (http://www.cerealsdb.uk.net/) were used for the identification of the full genomic sequence and subsequent reconstruction of the gene structure. The BLAST algorithm parameters were set as default.

**Table 2:** List of identified frost tolerance candidate gene sequences.

Candidate gene	Gene	Species	Cultivar	Accession	Type	Citation
*Cbf1*	*TaCBF1*	Triticum aestivum	Winoka	AF376136	Gene/CDS	[72]
*Cbf4*	*TmCBF4*	Triticum monococcum	n.a	AY951945	Gene/CDS	[73]
*Cbf5*	*TmCBF5*	Triticum monococcum	n.a	AY951947	Gene/CDS	[73]
*Cbf7*	*TmCBF7*	Triticum monococcum	DV92	AY785904	Gene/CDS	[26]
*Cbf8*	*HvCBF8*	Hordeum vulgare	Tremois	DQ445252	Gene/CDS	[25]
*Cbf10*	*TmCBF10*	Triticum monococcum	n.a	AY951950	Gene/CDS	[73]
*Cbf13*	*TmCBF13*	Triticum monococcum	n.a	AY951951	Gene/CDS	[73]
*Cbf14*	*TmCBF14*	Triticum monococcum	n.a	AY951948	Gene/CDS	[73]
*Cbf15*	*TaCBF15*	Triticum aestivum	Norstar	EF028765	Gene/CDS	[74]
*Cbf16*	*TmCBF16*	Triticum monococcum	G3116	EU076384	Gene/CDS	[75]
*Cbf17*	*TmCBF17*	Triticum monococcum	n.a	AY951945	Gene/CDS	[73]
*Cbf18*	*TmCBF18*	Triticum monococcum	n.a	AY951946	Gene/CDS	[73]
*Dhn1*	*HvDhn1*	Hordeum vulgare	Dicktoo	AF043087	Gene/CDS	[76]
*Dhn3*	*HvDhn3*	Hordeum vulgare	Dicktoo	AF043089	Gene/CDS	[76]
*Dhn4*	*HvDhn4*	Hordeum vulgare	Barke	BQ466915	EST	[77]
*Ice2*	*HvIce2*	Hordeum vulgare	Morex	DQ113909	Gene/CDS	[29]
*Vrn-A1*	*TaVRN-A1*	Triticum aestivum	Triple Dirk C Line	AY747600	Gene/CDS	[34]
*Vrn-B1*	*TaVRN-B1*	Triticum aestivum	Triple Dirk B Line	AY747603	Gene/CDS	[34]
*Vrn-D1*	*TaVRN-D1*	Triticum aestivum	Triple Dirk C Line	AY747606	Gene/CDS	[34]
*Vrn2*	*TaVRN2*	Hordeum vulgare	Dairokkaku	AY485977	partial CDS	[78]
*Vrn3*	*TaVRN3*	Triticum aestivum	Chinese Spring	DQ890162	Gene/CDS	[35]
*Cab*	*HvCab*	Hordeum vulgare	Barke	BQ465487	EST	[77]
*Dem*	*HvDem*	Hordeum vulgare	Barke	AL504294	EST	[79]
*Tacr7*	*HvTacr7*	Hordeum vulgare	Golden Promise	BQ659345	EST	[77]
*Ppd-A1*	*TaPpd-A1*	Triticum aestivum	Chinese Spring	DQ885753	Gene/CDS	[37]
*Ppd-B1*	*TaPpd-B1*	Triticum aestivum	Chinese Spring	DQ885757	Gene/CDS	[37]
*Ppd-D1*	*TaPpd-D1*	Triticum aestivum	Chinese Spring	DQ885766	Gene/CDS	[37]

### Reconstruction of intron-exon-structure and gene specific primer development

The reconstruction of the gene intron-exon-structure was performed using the internet platform ‘Spidey’ (http://www.ncbi.nlm.nih.gov/spidey/spideyweb.cgi) from NCBI, which allowsalignment of mRNA to genomic sequence. The intron/UTR regions sequences were used for primer development. The next step was the identification of the best hits to the three different wheat genomes on the IWGSC and/or the Bristol Wheat Genomics website via BLASTn. After collecting three homologue sequences of each targeted gene the gene structure was reconstructed for each one separately and then used for multiple alignments. Multiple alignments were constructed by using Sequencer 5.1 (Gene Codes Corporation, Ann Arbor, USA) and CLC Main Workbench 7.6 (CLC Bio, Aarhus, Denmark) software and visually inspected for unique stretches among three homologues. The polymorphisms between the three homologous genomes of each gene were detected and used for specific primer development. The primers were developed by using ‘Primer3’ (v. 0.4.0) [46, 47]. Parameters utilized for primer development were set to a maximal 3`stability of 50, primer size between 19 and 28 bp and primer melting temperature between 57° and 63°Celsius. The maximal fragment length was set up to 1200 bp, while optimal fragment length was 900 bp. Other parameters remained as default. Specificity of primers was based on two nucleotide differences within the primer binding site or one difference within the last seven nucleotides at the 3`end of the primer based on the analyses of the three homologue target sequences [48]. All primers were designed to bind locus specific sequences within the introns/UTR regions of selected genes. At least one primer of a primer pair had to be locus specific for single band amplification.

### PCR amplification and fragment analysis

Newly designed PCR primers were amplified in two different reaction volumes i.e. firstly, in a volume of 10 μl for functionality testing and chromosomal assignment, and secondly in a 20 μl reaction volume for re-sequencing. The PCR reactions comprised two different polymerases, FIREPol® DNA polymerase (Solis BioDyne, Tartu, Estonia), in a first round of testing, and MyTaq™ DNA polymerase (BIOLINE, Luckenwalde, Germany), in a second round of testing in case the FIREPol product was very weak, with 50 ng of genomic DNA. The master mix for one PCR reaction comprised 0.4 U FIREPol® DNA Polymerase, 1 x Buffer B, 2.5 mM MgCl2 (Solis BioDyne, Tartu, Estonia), 0.2 mM dNTPs (Fermentas, St. Leon-Rot, Germany) and 0.25 pmol primers (Microsynth, Balgach, Switzerland) or 0.4 U MyTaq™ DNA Polymerase, 1 x My Taq Reaction Buffer B (that comprised 1 mM dNTPs and 3 mM MgCl2) (BIOLINE, Luckenwalde, Germany) and 0.25 pmol primers. The fragment amplification was conducted in a thermal cycler GeneAmp® PCR System 9700 (Applied Biosystems, Darmstadt, Germany) under various PCR profiles (S2 Table). PCR fragments were separated by using agarose gel electrophoreses and analysed using the imaging system Gel Doc™ XR and the Quantity One® 1-D analysis software (4.6.2) (Bio-Rad, Hercules, USA).

### PCR fragment mapping by using NT- and deletion lines

All specific and single banded PCR fragments were assigned to chromosomes by using 21 nullisomic-tetrasomic (NT) lines [43] and by a set of 46 deletion-lines [44]. The information about chromosomal localisation of these gene specific amplicons was compared to published results. The map of specific PCR fragments was printed via LaTeX 4.4.1 software (freeware).

### *In silico* analysis of primer sub-genome specificity

A set of98 primers used for amplification of 65 PCR fragments with correct chromosomal localisation were in silico validated for sub-genome specificity by aligning to the draft sequence of wheat. The primers were aligned via Multiple Alignment using Fast Fourier Transform (MAFFT, http://www.ebi.ac.uk/Tools/msa/mafft/), CLC and Sequencher. Parameters for the Sequencher based alignment were as follows: clean data with minimum overlap of 19 nucleotides and minimum match percentage of 90%, while CLC and MAFFT parameters were as default. The differences between the sub-genome sequences and designed primers were manually inspected. Primers with sub-genome specificity were those having two or more differences in binding site or at least one difference at the last seven nucleotide bases at 3`end of primer.

### Re-sequencing of frost tolerance candidate genes and BLAST verification

Sequencing of PCR fragments was performed by Microsynth AG (Balgach, Switzerland) using the Sanger sequencing method [49]. First sequencing reactions were performed with primers used for amplification and if quality was lower than 70% an optimisation with redesigned oligos was conducted. Subsequently all fragment sequences were compared to reference sequences and/or candidate genes of related species by using NCBI MegaBlast function [50]. The results were limited to five hits, minimum expect threshold of e-^100^ and minimum identity of 85%. All other parameters remained as default. The haplotype diversity (Hd), the nucleotide diversity and the average number of nucleotide diversity in a set of 24 analysed wheat cultivars were calculated using the DnaSP 5.1 freeware software [51, 52].

## Results

Alignment of candidate gene sequences with corresponding genomic sequences retrieved from the International Wheat Genome Sequencing Consortium, the Bristol Wheat Genomics and NCBI allowed the identification of exon-intron splicing positions, and the identification of coding and non coding regions. Therefore, reconstruction of the intron-exon structure by using newly available genomic sequences is the basic step towards the development of gene specific primers in polyploid plants such as hexaploid wheat.

### Reconstruction of intron-exon-structure and development of gene specific primers

**Fig 1:**
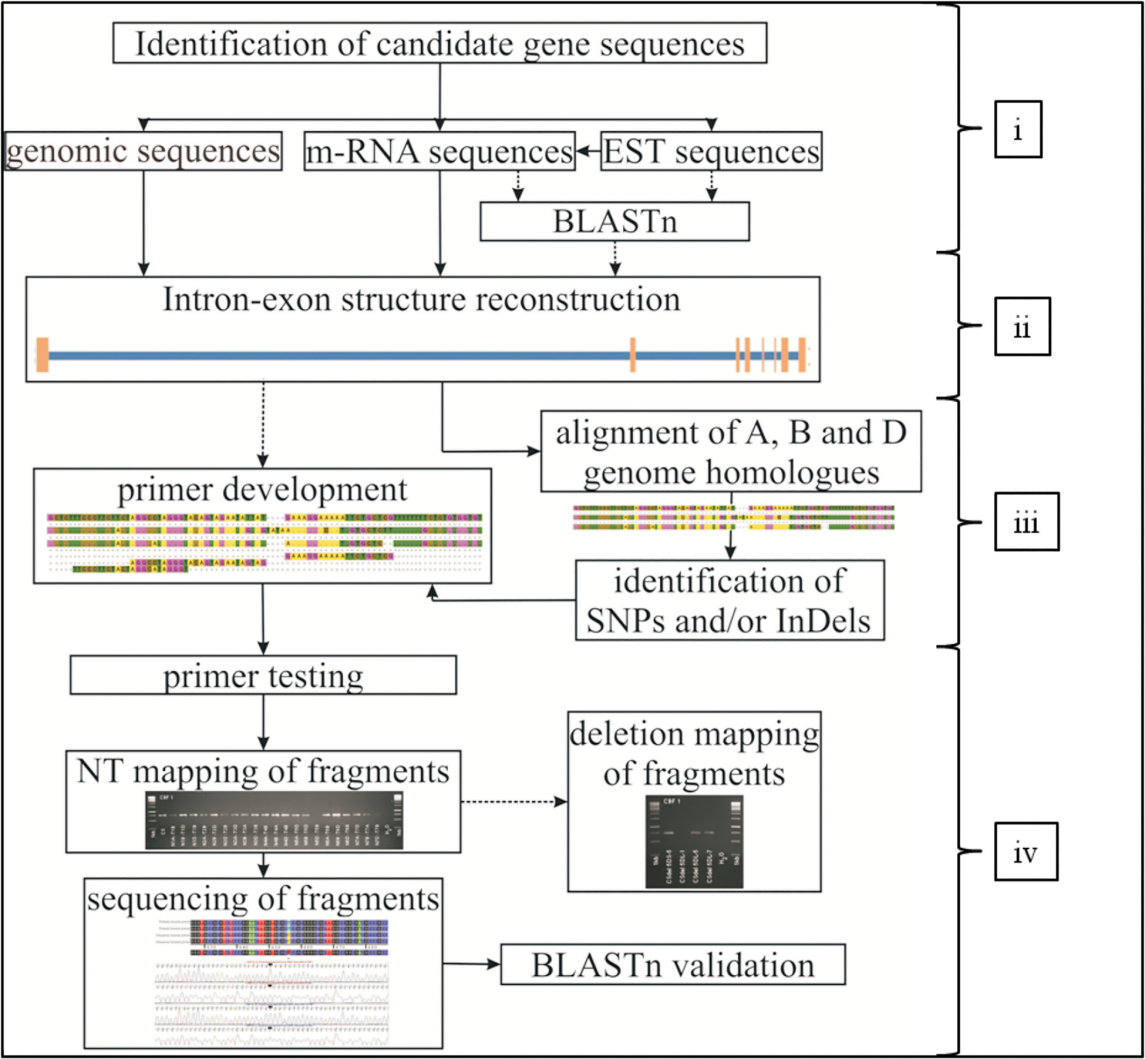
Workflow of development gene specific primers and PCR fragments in wheat. The method comprises four steps, i.e. (i) identification of genomic and coding sequences (CDS) of candidate genes, (ii) intron- and exon-structure reconstruction, (iii) identification of wheat A, B and D sub-genome sequences and primer development on sequence differences between the three sub-genomes, and (iv); primer and PCR fragment testing for functionality, correct size and localisation. The dashed lines show optional applications.

The workflow for the development of gene specific primers and validation regarding PCR specificity, chromosomal localisation and sequence homology contains four steps (Fig 1). In short, the procedure starts with collecting sequences of candidate genes, followed by the reconstruction of intron and exonstructure and sub-genome sequence identification, until primer development and PCR fragment testing. Functionality and correctness of PCR fragments were assessed by NT mapping, sequencing and BLASTing by using three databases, six tools (‘Spidey’, ‘Primer3’, BLASTn, BLASTx, CLC Main Workbench and Sequencer) and two cytological stocks of wheat.

**Fig 2:**
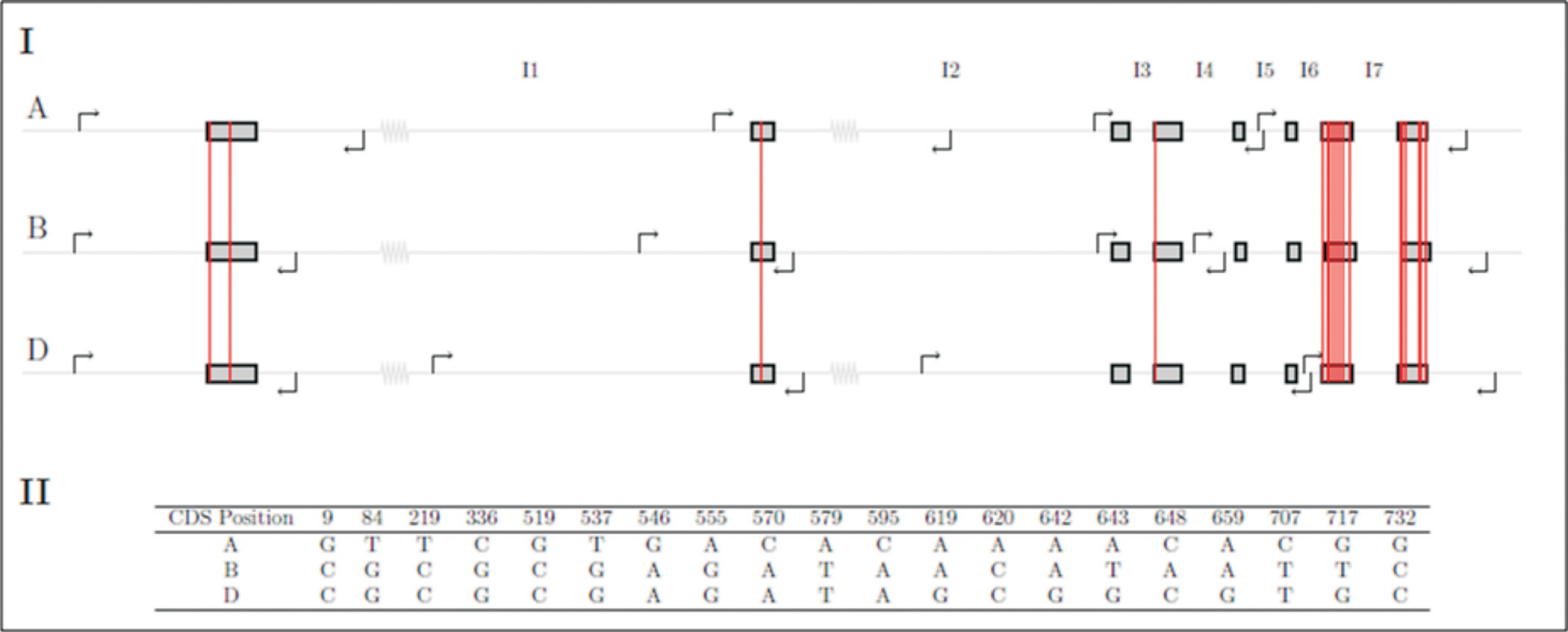
Example for an intron-exon structure, intron length differences, exon SNPs and primer position of the three copies of the *Vrn1* gene. (I) A, B and D on the left border stand for the three different wheat sub-genomes and I1 to I7 on the top for the seven introns of *Vrn1*. The arrows which bend to the right are forward primers and the arrows which bend to the left are reverse primers. The red vertical lines show SNPs between the three gene copies. A I1 has a length of 8518 bp, B I1 2821 bp, D I1 8625bp, A I2 1475 bp, B I2 1246 bp, D I2 1504 bp, A I3 90 bp, B I3 92 bp, D I3 90 bp, A I4 192 bp, B I4 196 bp, D I4 188 bp, A I5 152 bp, B I5 156 bp, D I5 156 bp, A I6 93 bp, B I6 91 bp, D I6 91 bp, A I7 166 bp, B I7 168 bp and D I7 168 bp. (II) This figure shows the SNPs between the three sub-genomes and their coding sequences (CDS) position.

For all of the 27 candidate genes we were able to re-construct the gene structure or at least a part of it. A set of 119 PCR products was obtained from 157 primers pairs designed in this study. 13 of them have recently been published in Keilwagen et al. [53]. Additional 12 primers from literature were used for the amplification of targeted genes. By combining the primers from this study and the 12 primers from literature a total of 169 primers were analysed. As an example the reconstruction of the three copies of the *Vrn1* gene structure, primer positions, intron length differences and exon SNPs are shown in Fig 2.

### Testing primers for specificity and chromosomal assignment of PCR products

In total, a set of 169 primers representing 119 PCR products from 27 candidate genes was tested for functionality and specificity. A set of 86 primer combinations from 23 candidate genes showed single band amplification (72.27%).

**Fig 3:**
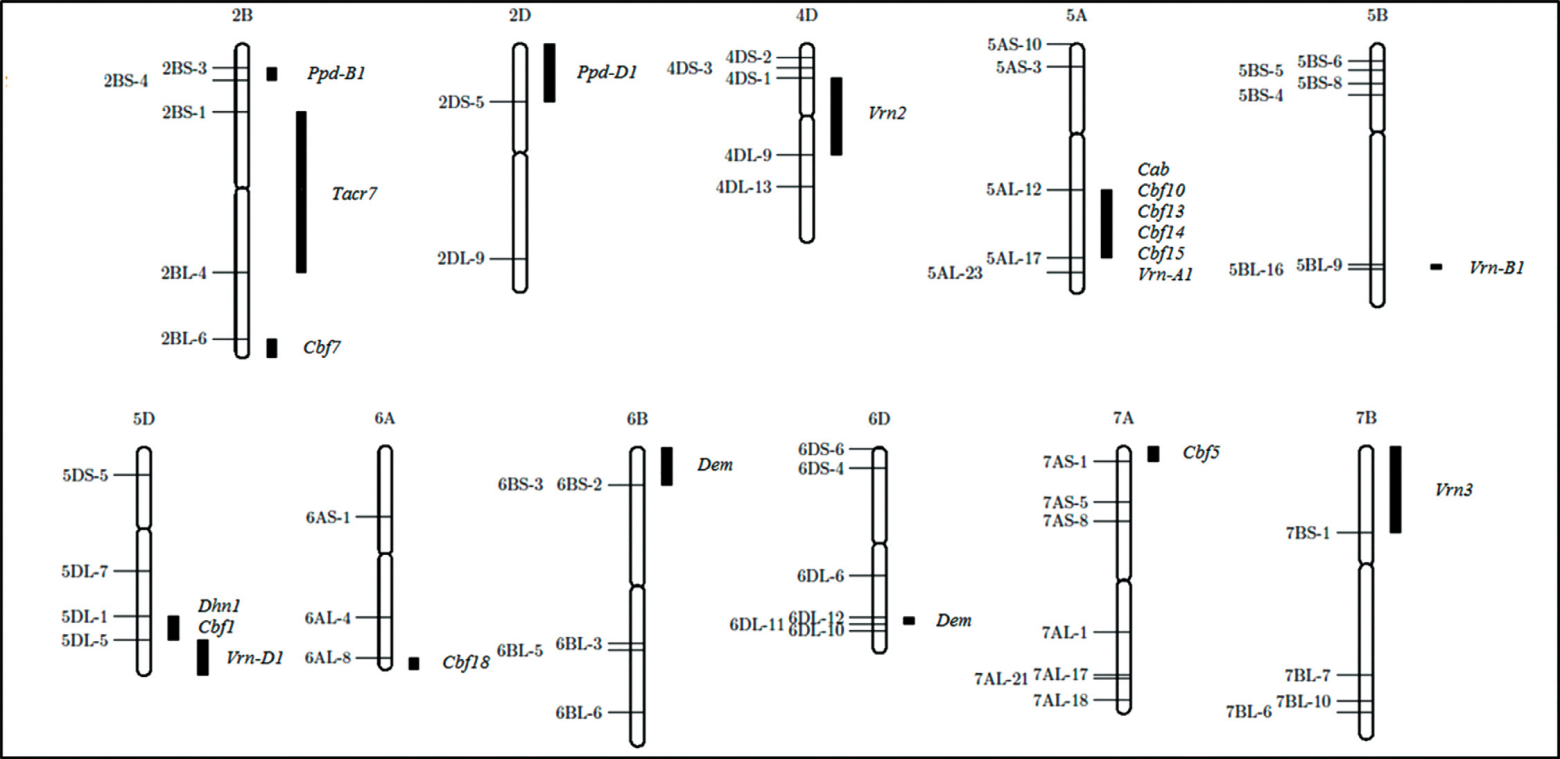
Map of gene specific PCR fragments by using wheat NT- and deletion lines. In this figure only wheat chromosomes are shown harbouring mapped PCR fragments. The white bar is the chromosome, the constriction symbolised the centromere, on the left side of chromosomes deletion break points are listed and the black bars are the regions of mapped PCR fragments with appended candidate gene.

Chromosomal localisation via Nulli-tetrasomic (NT)-lines of Chinese Spring [43] of a set of 86 single band PCR amplicons revealed that 65 fragments were located on expected chromosomes according to the literature. Out of these 65 fragments, six were products of combination of already published and newly designed primers. The remaining 19 fragments showed an incorrect localisation (literature vs. NT-lines) or no localisation was possible as all NT-lines showed a fragment. Correctly assigned amplicons originated from 19 genes and were located on 11 wheat chromosomes (Table 3, Fig 3). A set of 10 out of 19 analysed genes were located on wheat chromosome group 5, out of 119 PCR fragments 65 single bands were correctly localised. That is equivalent to a success rate of 54.6%. These 65 amplicons represent 19 frost tolerance genes, are gene specific and were therefore selected for further studies (Table 4, S2 Table).

**Table 3:** Overview of chromosome localisation of candidate genes (PCR fragment) via NT-lines, deletion-lines and literature.

Gene	NT-lines	PCR signal present via deletion-lines	Deletion-line localisation section	Literature location	Reference
*Cbf1*	5D	5DS-5; 5DL-5,-7	proximal from 5DL-5 and distal from 5DL-1	n.a	
*Cbf5*	7A	7AL-1	distal on short arm from 7AS-1	7Am	[73]
*Cbf7*	2B	2BS-1,-3,-4	distal on long arm from 2BL-6	n.a	
*Cbf10*	5A	5AS-3,-10; 5AL-17,-23	proximal from 5AL-12 and 5AL-17	5Am	[73]
*Cbf13*	5A	5AS-3,-10; 5AL-17,-23	proximal from 5AL-12 and 5AL-17	5Am	[73]
*Cbf14*	5A	5AS-3,-10; 5AL-17,-23	proximal from 5AL-12 and 5AL-17	5Am	[73]
*Cbf15*	5A	5AS-3,-10; 5AL-17,-23	proximal from 5AL-12 and 5AL-17	5Am	[73]
*Cbf18*	6A	6AS-1	distal on long arm from 6AL-8	6Am	[73]
*Dhn1*	5D	5DS-5; 5DL-5	proximal from 5DL-5 and distal from 5DL-1	5H	[80]
*Vrn-A1*	5A	5AS-3,-10; 5AL-17,-23	proximal from 5AL-12 and 5AL-17	5A	[81]
*Vrn-B1*	5B	5BS-4,-5,-6,-8; 5BL-16	proxiaml from 5BL-16 and distal from 5BL-9	5B	[81]
*Vrn-D1*	5D	5DS-5; 5DL-7	distal on long arm from 5DL-5	5D	[81]
*Vrn2*	4D	everywhere (4DS-1,-2;-3; 4DL-9,-13)	proximal from 4DS-1 and 4DL-9	5AmL	[82]
*Vrn3*	7B	7BL-7;-6;-10	distal on short arm from 7BS-1	7BS	[35]
*Cab*	5A	5AS-3,-10; 5AL-17,-23	proximal from 5AL-12 and 5AL-17	5HL	[55]
*Dem*	6B/6D	6BS-2; 6BL-3,-5,-6; 6DS-4,-6; 6-DL 10	distal on short arm from 6BS-3 and proximal from 6DL-11 and distal from 6DL-12	6HL	[55]
*Tacr7*	2B	everywhere (2BS-1,-3,-4; 2BL-6)	proximal from 2BS-1 and 2BL-6	2HL	[55]
*Ppd-B1*	2B	2BS-3; 2BL-6	proximal from 2BS-1 and distal from 2BS-4	2B	[83–85]
*Ppd-D1*	2D	2DL-9	distal on short arm from 2DS-5	2D	[84, 85]

The table shows the analysed frost tolerance candidate gene, their chromosomal localisation and fine mapping via NT and deletion-lines. The column deletion-line localisation section shows the approximate chromosomal position of respective genes based on deletion break points.

**Table 4:** Primer sequences used for amplification of candidate genes.

Fragment	Forward primer name	Forward primer sequence (5`- 3`)	Reverse primer name	Reverse primer sequence (5`- 3`)
Cbf1	AF376136_s1	TTTTTGACGCTGCAACTGAT	AF376136_as709	TTTACCGAGGGAGTAGTTTCCA
Cbf5	TmCBF5_F *^2^	CGATGCAAAGTGTGCAATTC	AY951947_as1691	ACTAGCTCATGCGAATATGGTGT
Cbf7	AY785904_s4	TTCTAGTCCACCTAGCTACAGGC	AY785904_as926	CACTAGCAAAGCAATTCATGAGC
Cbf10	AY951950_s1522	ACATCTCACACACTCCACAGATG	Cbf4B_R *^3^	GCAGAATCGGCTACAAGCTCCAG
Cbf13	Cbf5_F *^3^	CAGAGCAGAATCAGATGGGGAATC	AY951951_as1964	GCTAAGCTCACACTCCTCGATAA
Cbf14	AY951948_s_565	TAAACTCGCTGCTTAATTACCCC	AY951948_as_1312	ATATTTGGTGGAACAGAAGCAGA
	AY951948_s_528	CAGCATCCATCTCTCTCAAATCT	AY951948_as_1299	CAGAAGCAGAGAAACCGTCTAAA
	AY951948_s_565	TAAACTCGCTGCTTAATTACCCC	AY951948_as_1299	CAGAAGCAGAGAAACCGTCTAAA
	AY951948_s_528	CAGCATCCATCTCTCTCAAATCT	AY951948_as_1312	ATATTTGGTGGAACAGAAGCAGA
Cbf15	EF028765_s_90	ACCGACCACCTGCAGTACC	EF028765_as_875	TTGTTCCATGCATAGAGTCAAAG
Cbf18	AY951946_s400	CGTATAAATACGCACACGCACTA	AY951946_as1445	ACATGGTGGAGGGATCTTTTTAT
Dhn1	ScDhn1_F *^1^	CCACGTAGCACGCACGCTGT	AF043087_as1808	TCGGAACATAGAGAAGACACACA
Vrn-A1b	VRN1-A_F *^4^	GAAAGGAAAAATTCTGCTCG	AY747600_as1083	GATTACCGTCTTAACCCTTCCAC
Vrn-A1c	AY747600_s9072	CATGAAACAACGCATTACAGAAA	AY747600_as10169	CAGATAGAACTGGTTGGATCCCT
Vrn-A1d	AY747600_s_10698	TTTCTGTCATTGTTCCTTCCTGT	AY747600_as_11318	CAAGCTAAGGCTTCATGACAAGT
	AY747600_s_10718	TGTCCCACCCAAAGTTAGTAATG	AY747600_as_11390	AACGATGTAATGAGGTTACGTGC
	AY747600_s_10698	TTTCTGTCATTGTTCCTTCCTGT	AY747600_as_11390	AACGATGTAATGAGGTTACGTGC
	AY747600_s_10718	TGTCCCACCCAAAGTTAGTAATG	AY747600_as_11318	CAAGCTAAGGCTTCATGACAAGT
Vrn-A1e	AY747600_s_11297	CTTGTCATGAAGCCTTAGCTTGT	AY747600_as_12066	GCTGCAGCTTGCTACTTTACTCT
	AY747600_s_11297	CTTGTCATGAAGCCTTAGCTTGT	AY747600_as_12099	AAACTGAGGTGGACAAAGTGAAA
Vrn-B1b	AY747603_s18	AGGCCTAGGGTACAGTAGAATAGTAG	AY747606_as820	CAAACGGAATCAACCAAACAG
Vrn-B1c	AY747603_s3097	TCTGAGCAGAATTATACTTACCTTGC	AY747606_as9488	AGATCATCTGATATCGGCAAAAA
Vrn-B1d	AY747603_s_4783	CCTTCCTGTTCCACTCAAAGTTA	AY747603_as_5249	TTTTTAACTGTGAAGAGCATATGACTAA
Vrn-B1e	AY747603_s5134	AAACAAGAAAAACACTTGCAGAGA	AY747603_as6211	ATTACATGGTAAATTGAGCCCAG
Vrn-D1b	AY747606_s6	TTCCCTTCTACTAGGCATAGGGT	AY747606_as820	CAAACGGAATCAACCAAACAG
Vrn-D1c	AY747606_s8129	GTGTTGGTAGAAGGCTAGAAGCA	AY747606_as9488	AGATCATCTGATATCGGCAAAAA
Vrn-D1d	AY747606_s10179	GACCTCACGCCAATTTTTGT	AY747606_as11608	TACGAAACAATTTAGACCGGTTG
Vrn-D1e	AY747606_s11586	CAACCGGTCTAAATTGTTTCGTA	AY747606_as12291	TTAATTCACATAAACAACATCCCACTA
Vrn2a	AY485977_s306	AAACAAGCAAACGTTGGAGTTAG	AY485977_as1282	AATAAGCAATTTCCTGATGCAAA
Vrn2b	AY485977_s_1542	CAACACTGAATGAAAATGGATCA	AY485977_as_1985	GAACCATCCGAGGTGAAGTTTA
	AY485977_s_1542	CAACACTGAATGAAAATGGATCA	AY485977_as_1972	TGAAGTTTACTAGGATCATGGGG
	AY485977_s_1439	CCATAGAGCAATTGAGTTTGGAC	AY485977_as_1972	TGAAGTTTACTAGGATCATGGGG
Vrn2a/b	AY485977_s306	AAACAAGCAAACGTTGGAGTTAG	AY485977_as_1972	TGAAGTTTACTAGGATCATGGGG
Vrn3a	DQ890162_s_1430	AAGGAGTACTAGAGCGGCGAG	DQ890162_as_1915	TGTGGTGAGCACTTTCAGAGATA
	DQ890162_s_1552	TTCCTCAATTCACAGCTTACTCC	DQ890162_as_1915	TGTGGTGAGCACTTTCAGAGATA
Vrn3b	DQ890162_s2159	TCTTAAATACTCTCTCCGTCCGA	DQ890162_as3153	AAGCCATTGATCTAGGGTTCAC
	DQ890162_s2396	GAAGTACACTTATTCGTGGACGG	DQ890162_as3153	AAGCCATTGATCTAGGGTTCAC
Vrn3a/b	DQ890162_s_1552	TTCCTCAATTCACAGCTTACTCC	DQ890162_as3153	AAGCCATTGATCTAGGGTTCAC
Cab b	contig22616_s209	TTTTGCGAAAGCACACTTATACA	contig22616_as938	GAAGCATCGCCAGCTATAAATAC
Cab d	contig22616_s209	TTTTGCGAAAGCACACTTATACA	contig22616_as828	CAGTTGCAGCAGAGAGATTCTT
Dem	CD937801_s29	ATACCATCGGCAACTCCTCTG	contig1013618_as520	CCATTATGGATAGCGAAATTTGA
Tacr7 b	contig4120743_s26	CAACCAAAACTCGCCTATAAAAG	contig2688312_as455	AATCGGAGAGGAAGCTCTCTTTA
Tacr7 c	contig4120743_s271	CGAGGAGAAGGTTTGGGGTT	contig2688312_as455	AATCGGAGAGGAAGCTCTCTTTA
Tacr7 d	BJ246882_s196	GTCGGCGAGGAGAAGGTTTT	contig2688312_as455	AATCGGAGAGGAAGCTCTCTTTA
Ppd-B1c	DQ885757_s11028	TCCTTCCAGCTTACTAGTGCATC	DQ885757_as11954	ATCACCTGGAAAACATATTGGAA
Ppd-B1d	DQ885757_s11883	AACTGAACCAAAAGCCTGCTACT	DQ885757_as12453	GTACCTTGCAAAGAATGAAAACG
Ppd-B1e	DQ885757_s_12390	CCTTTGTGAATCCTTAAATCATCC	DQ885757_as_13162	AACAGAGAACAAACGAAATCGG
Ppd-B1f	DQ885757_s_13184	GGGCTTATTCATGATAGCTGATG	DQ885757_as_13562	ATCGACTCCGCACTTCTACTATG
	DQ885757_s_13148	CGTTTGTTCTCTGTTCTTCGTTT	DQ885757_as_13625	ACCGTTACACAGGTTCAGACATT
	DQ885757_s_13184	GGGCTTATTCATGATAGCTGATG	DQ885757_as_13625	ACCGTTACACAGGTTCAGACATT
	DQ885757_s_13148	CGTTTGTTCTCTGTTCTTCGTTT	DQ885757_as_13562	ATCGACTCCGCACTTCTACTATG
Ppd-D1 Prom	DQ885766_s3601 ^†^ ^6^	CTTGTCCAACTCCCAATCTAGTG	DQ885766_as4689 ^†^ ^6^	TCCTCCCCTGTTTCTTTTTACTC
	DQ885766_s4578	TCGTCCATCCAAAGATACTGATT	DQ885766_as5712 ^†^ ^6^	AGTACGCTGCCGTGAGTAATAAT
	DQ885766_s4450 ^†^ ^6^	CATACTCCCTCCGTTTCTTCTTT	DQ885766_as5712 ^†^ ^6^	AGTACGCTGCCGTGAGTAATAAT
	DQ885766_s4578	TCGTCCATCCAAAGATACTGATT	DQ885766_as5700	TGAGTAATAATCGAACCTCGGTC
Ppd-D1a	DQ885766_s5689	ATTATTACTCACGGCAGCGTACT	DQ885766_as6299 ^†^ ^6^	TACTGAAACATTTTAGGGCCAAG
	DQ885766_s5677 ^†^ ^6^	GACCGAGGTTCGATTATTACTCA	DQ885766_as6299 ^†^ ^6^	TACTGAAACATTTTAGGGCCAAG
Ppd-D1a2	DQ885766_s5766 ^†^ ^6^	CAACATGTTTCCTCTTGGAGC	DQ885766_as6535 ^†^ ^6^	GAACAGAGTCAAACACCATCAGA
Ppd-D1b	DQ885766_s6298	TATCAGGTTCATTTGCTTCAGTG	DQ885766_as7002 ^†^ ^6^	ATGGACAAATTGACCTCTAGTGC
	DQ885766_s6277 ^†^ ^6^	CTTGGCCCTAAAATGTTTCAGTA	DQ885766_as7002 ^†^ ^6^	ATGGACAAATTGACCTCTAGTGC
	DQ885766_s6277 ^†^ ^6^	CTTGGCCCTAAAATGTTTCAGTA	DQ885766_as6963	GCCATTCAGTTTTATCTAGCTTCC
Ppd-D1c	DQ885766_s7244	TGACAAGTATCTGCATCTGAACC	DQ885766_as8033 ^†^ ^6^	GATTCGCAAAGGACACTGATATT
Ppd-D1d	DQ885766_s6939 ^†^ ^6^	GGAAGCTAGATAAAACTGAATGGC	DQ885766_as8033 ^†^ ^6^	GATTCGCAAAGGACACTGATATT
Ppd-D1e	DQ885766_s8011 ^†^ ^6^	AATATCAGTGTCCTTTGCGAATC	Ppd-D1exon8_R1 *^5^	gtctaaatagtaggtactagg
Ppd-D1 3`UTR	DQ885766_s8771	CTGCTCTCTGTTCTTGGTTTCAT	DQ885766_as9720	ACCTCCCTGACGAAAAGCTC

Primer names with ^†^ are developed in course of this work but published from Keilwagen et al. [53].

Primer names with * as already published were used in combination with primers with ^†^ and without labels.

^1^[86]

^2^[73]

^3^[87]

^4^[81]

^5^[37]

^6^ [53]

Furthermore, a set of 40 amplicons was physically assigned using a set of 46 available deletion-lines [44] (Fig 3, Table 3). All six genes, which are localised on chromosome 5A via NT-lines, are map to a large cluster between sector AL-12 and AL-17 on the long arm of chromosome 5.

### *In silico* analysis of primer sub-genome specificity

The draft sequence of wheat and related species allows detailed *in silicio* analysis of oligos used in this study by doing simple BLAST comparison. Out of 98 oligos that were used for the amplification of 65 PCR fragments, 54 turned out to be specific to one sub-genome, 21 specific to two sub-genomes, and 14 were unspecific. For 9 oligos the comparison could not be performed due to non availability of sub-genome sequences (S2 Table). 57 out of 65 amplicons comprise at least one sub-genome specific primer. For five PCR fragments (Cbf5, Dhn1, Cab b, Cab d and Dem) no wheat sub-genome sequences could be identified. Both primers of PCR fragments Cbf7, Ppd-B1f and Ppd-D1b showed no-specificity to one sub-genome in reference to Wu et al. [48]. Nevertheless, all three fragments showed single bands and correct chromosome localisation via NT-lines (S1 Fig). The primer sequences of Ppd-B1f and Ppd-D1b were derived from a specific sub-genome. At least one of the primers showed one or more differences to corresponding regions on the chromosomes in alignments with the other two sub-genomes. Special cases are the primers of fragment Cbf7. The forward primer has no sub-genome specificity and the reverse primer is specific to sub-genomes A and B (S3 Table).

### Re-sequencing of genes involved in frost tolerance and homology validation via BLAST

Five out of 40 amplicons revealed a presence/absence polymorphisms (dominant) and were therefore not sequenced. These five dominant markers were directly used for genotyping of a *Ppd-D1* deletion in the promoter and a transposable element (TE) in intron1 [54]. One PCR fragment (Cbf7) could not be sequenced due to very low quality. Finally, 34 amplicons, representing 18 frost tolerance genes, were selected for sequencing and all 34 obtained sequences were compared to retrieved gene models via MegaBlast. In case of *Tacr7*, Kocsy et al. [55] identified BQ659345 of *Hordeum vulgare* as reference, but the fragments of 24 sequenced genotypes of *Tacr7* do not exceed an identity of 84% to the published wheat reference sequence L28093 for *Tacr7*[31]. The best BLAST hit of the 24 sequences is still the initial barley sequence BQ659345 with an identity of 92.3%, but the second best barley BLAST hit of 91.8% identity is to X97916, the barley low temperature gene 14.1 abbreviated as *blt14*.*1*. The rest of 33 BLAST results show very high identities from 88.8% to 100% to initial gene sequences (Table 5).

**Table 5:** BLASTn results of sequenced PCR fragments versus NCBI nucleotide collection (nr/nt) and NCBI candidate gene reference EST.

Gene	PCR fragment	Database	Subject Seq-id (ID of the database hit)	Percentage of identical matches	Expectation value (E-value)	Bit score	Subject discribtion
*Cab*	Cab b	EST	gi|21273269|gb|BQ465487.1|	93,71	0	643	HU03M14r HU Hordeum vulgare subsp. vulgare cDNA clone HU03M14 5-PRIME, mRNA sequence.
*Cbf1*	Cbf1	nucleotide	gi|17226800|gb|AF376136.1|	100	0	1279	Triticum aestivum putative CRT/DRE-binding factor (CBF1) mRNA, complete cds
*Cbf5*	Cbf5	nucleotide	gi|404415276|gb|JN987194.1|	100	0	1519	Triticum aestivum AP2 domain CBF protein (CBFII) mRNA, CBFII-5.4 allele, complete cds
*Cbf10*	Cbf10	nucleotide	gi|404415286|gb|JN987199.1|	99,53	0	1548	Triticum aestivum AP2 domain CBF protein (CBFIIIc) mRNA, CBFIIIc-10.1 allele, complete cds
*Cbf13*	Cbf13	nucleotide	gi|404415320|gb|JN987217.1|	100	0	1493	Triticum aestivum AP2 domain CBF protein (CBFIIIc) pseudogene, CBFIIIc-13.1c allele, complete sequence
*Cbf14*	Cbf14	nucleotide	gi|158999375|gb|EU076382.1|	99,01	0	902	Triticum monococcum CBF14 gene, complete cds
*Cbf15*	Cbf15	nucleotide	gi|404415321|gb|JN987218.1|	100	0	1325	Triticum aestivum AP2 domain CBF protein (CBFIIId) gene, CBFIIId-15.2b allele, complete cds
*Cbf18*	Cbf18	nucleotide	gi|63098599|gb|AY951946.1|	94,92	0	1559	Triticum monococcum CRT/DRE binding factor 18 (CBF18) gene, complete cds
*Dem*	Dem b	nucleotide	gi|241986478|dbj|AK333739.1|	97,37	0	713	Triticum aestivum cDNA, clone: WT008_O03, cultivar: Chinese Spring
		EST	gi|12030509|emb|AL504294.1|	90,37	7,00E-128	466	AL504294 Hordeum vulgare Barke roots Hordeum vulgare subsp. vulgare cDNA clone HW04N07 5', mRNA sequence.
*Dhn1*	Dhn1	nucleotide	gi|59894280|gb|AY895879.1|	88,79	3,00E-176	625	Hordeum vulgare subsp. spontaneum voucher NPGS PI 559556 dehydrin 1 (Dhn1) gene, partial cds
*Ppd-B1*	Ppd-B1c	nucleotide	gi|456359289|dbj|AB745620.1|	100	0	1679	Triticum turgidum subsp. pyramidale Ppd-B1 gene for pseudo-response regulator, complete cds, strain: KU-9882
	Ppd-B1d	nucleotide	gi|456359289|dbj|AB745620.1|	99,82	0	1009	Triticum turgidum subsp. pyramidale Ppd-B1 gene for pseudo-response regulator, complete cds, strain: KU-9882
	Ppd-B1e	nucleotide	gi|456359289|dbj|AB745620.1|	99,3	0	1297	Triticum turgidum subsp. pyramidale Ppd-B1 gene for pseudo-response regulator, complete cds, strain: KU-9882
	Ppd-B1f	nucleotide	gi|383215299|gb|JF946486.1|	99,74	0	693	Triticum aestivum transposon TREP 3040_Harbinger, complete sequence; pseudo-response regulator (Ppd-B1) gene, Ppd-B1a allele, complete cds; and retrotransposon Gypsy TREP 3457_Danae, complete sequence
*Ppd-D1*	Ppd-D1d	nucleotide	gi|395759126|dbj|AB646977.1|	99,91	0	1965	Triticum aestivum PRR gene for pseudo-response regulator, complete cds, allele: Ppd-D1b.2
	Ppd-D1e	nucleotide	gi|395759124|dbj|AB646976.1|	100	0	1731	Triticum aestivum PRR gene for pseudo-response regulator, complete cds, allele: Ppd-D1a.1
	PpD1 3`UTR	nucleotide	gi|118638641|gb|DQ885766.1|	100	0	1629	Triticum aestivum cultivar Chinese Spring chromosome 2D pseudo-response regulator (PRR) gene, complete cds
*Tacr7*	Tacr7 b	nucleotide	gi|1418967|emb|X97916.1|	91,75	0	778	H.vulgare blt14.1 gene
		EST	gi|21800478|gb|BQ659345.1|	92,34	5,00E-180	640	HD01A06w HD Hordeum vulgare cDNA clone HD01A06 3-PRIME, mRNA sequence.
*Vrn2*	Vrn2a	nucleotide	gi|211593611|gb|FJ173824.1|	91,07	6,00E-149	534	Triticum turgidum retrotransposon Wilma, partial sequence; and ZCCT2-B2b (VRN2) gene, complete cds
	Vrn2b	nucleotide	gi|45390737|gb|AY485977.1|	92,57	0	1120	Hordeum vulgare cultivar Dairokkaku ZCCT-Ha (VRN2) gene, partial cds
*Vrn3*	Vrn3a	nucleotide	gi|117168399|gb|DQ890162.1|	100	0	1825	Triticum aestivum cultivar Chinese Spring VRN3 (vrn-B3) gene, complete cds
	Vrn3b	nucleotide	gi|117168399|gb|DQ890162.1|	100	0	1801	Triticum aestivum cultivar Chinese Spring VRN3 (vrn-B3) gene, complete cds
*Vrn-A1*	Vrn-A1b	nucleotide	gi|383215288|gb|JF965395.1|	100	0	1829	Triticum aestivum cultivar Claire VRN-A1 (VRN-A1) gene, complete cds
	Vrn-A1c	nucleotide	gi|383215290|gb|JF965396.1|	100	0	1995	Triticum aestivum cultivar Malacca VRN-A1 (VRN-A1) gene, complete cds
	Vrn-A1d	nucleotide	gi|383215290|gb|JF965396.1|	100	0	1109	Triticum aestivum cultivar Malacca VRN-A1 (VRN-A1) gene, complete cds
	Vrn-A1e	nucleotide	gi|383215292|gb|JF965397.1|	100	0	1279	Triticum aestivum cultivar Hereward VRN-A1 (VRN-A1) gene, complete cds
*Vrn-B1*	Vrn-B1b	nucleotide	gi|384371844|gb|HQ130483.2|	100	0	1459	Triticum aestivum cultivar Diamant2 Vrn-B1 (Vrn-B1) gene, Vrn-B1-a allele, promoter region and complete cds
	Vrn-B1c	nucleotide	gi|384371844|gb|HQ130483.2|	99,82	0	1016	Triticum aestivum cultivar Diamant2 Vrn-B1 (Vrn-B1) gene, Vrn-B1-a allele, promoter region and complete cds
	Vrn-B1d	nucleotide	gi|384371844|gb|HQ130483.2|	100	0	867	Triticum aestivum cultivar Diamant2 Vrn-B1 (Vrn-B1) gene, Vrn-B1-a allele, promoter region and complete cds
	Vrn-B1e	nucleotide	gi|58423007|gb|AY747604.1|	100	0	1969	Triticum aestivum cultivar Triple Dirk C line VRN-B1 (VRN-B1) gene, complete cds
*Vrn-D1*	Vrn-D1b	nucleotide	gi|58423011|gb|AY747606.1|	100	0	1328	Triticum aestivum cultivar Triple Dirk C line VRN-D1 (VRN-D1) gene, complete cds
	Vrn-D1c	nucleotide	gi|58423011|gb|AY747606.1|	100	0	1701	Triticum aestivum cultivar Triple Dirk C line VRN-D1 (VRN-D1) gene, complete cds
	Vrn-D1d	nucleotide	gi|58423011|gb|AY747606.1|	99,16	0	1714	Triticum aestivum cultivar Triple Dirk C line VRN-D1 (VRN-D1) gene, complete cds
	Vrn-D1e	nucleotide	gi|58423011|gb|AY747606.1|	100	0	1327	Triticum aestivum cultivar Triple Dirk C line VRN-D1 (VRN-D1) gene, complete cds

In 12 genes out ofa set of 18 sequenced candidate genes represented by 16 unique PCR amplicons, differences between the 24 genotypes were determined, revealing a high level of polymorphimsof 66.67%. The number of polymorphic sites ranged from 1 to 37, the haplotypes (h) from two to three, the haplotype diversity (Hd) from 0.08 to 0.61 and the nucleotide diversity (π) from 0.00008 to 0.00757 (Table 6).

**Table 6:** Nucleotide polymorphisms of coding and noncoding candidate gene regions.

Gene	No. accessons	No. of bp	No. of polymorphic sites	Percentage polymorphism	h	Hd	k	π	k (i)	π (i)
*CBF5*	23	824	2	0,24	3	0,49	0,95	0,00115	0,44	0,00054
*CBF10*	23	776	2	0,26	2	0,47	0,95	0,0012	n/a	n/a
*CBF13*	23	773	4	0,52	2	0,47	1,42	0,00193	0,95	0,00123
*CBF14*	22	1184	6	0,51	3	0,48	1,91	0,00163	0,46	0,00038
*CBF15A*	24	755	7	0,93	2	0,49	2,94	0,00395	0,49	0,00065
*CBF18*	22	951	37	3,89	2	0,09	3,09	0,00328	0,27	0,00029
*Vrn-A1*	24	2954	9	0,30	2	0,08	0,42	0,00014	0,33	0,00011
*Vrn-D1*	23	3093	1	0,03	2	0,24	0,24	0,00008	n/a	n/a
*Vrn3*	24	1566	1	0,06	2	0,52	0,52	0,00033	n/a	n/a
*Cab*	24	707	13	1,84	3	0,61	5,18	0,00757	1,26	0,00179
*Ppd-B1*	24	3971	2	0,05	3	0,36	0,37	0,00009	n/a	n/a
*Ppd-D1*	24	2642	1	0,04	2	0,23	n/a	n/a	0,23	0,00009

h haplotypes

Hd haplotype diversity

k average number of nucleotide differences

π nucleotide diversity

(i) InDel

The results of the workflow for locus specific primer development presented in this paper are very promising. The main workflow step is the identification of sub-genome sequences and the design of primers on sub-genome sequence differences. This is the essential step of this workflow and is crucial for the success of this approach. The primer amplification test for single bands and the fragment mapping via NT-lines are a simple way to verify locus specificity. The sequencing of selected locus specific amplicons and the BLAST analysis of these fragment sequences versus initial data bases is the last step of safe-guarding the correct amplification. The results of this BLAST search showed no critical differences to the initially selected sequences.

## Discussion

New bioinformatic platforms and data bases containing recent genomics data are a powerful resource for the development of tools for molecular plant breeding.

### Gene specific primer development and chromosomal assignment of specific PCR fragments by using NT- and deletion lines

The rapid progress in sequencing of plant genomes leads to the accumulation of whole genome sequence data,allowing the fast development of locus/genome specific markers in complex plant genomes (e.g. wheat) with a high success rate. Up to now, high homology of the hexaploid wheat genome hampered the success in gene specific primer development. Gene structure is important for marker development, because wheat introns have more sequence differences between the homologous chromosomes than exons [56, 57]. Therefore, gene structure reconstruction and comparison of homologue sequences by using three genomes facilitate an improved development of molecular markers as well as re-sequencing of targeted genes/loci.

### Specificity of developed primers

**Fig 4:**
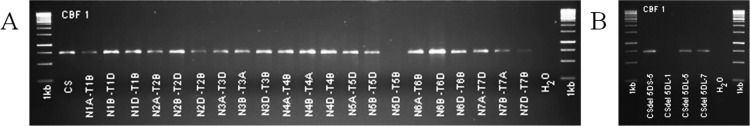
Example of fragment localisation from *Cbf1* via NT- and deletion-lines. (A) The missing PCR fragment on NT-line N5D-T5B indicated the location on wheat chromosome 5D. (B) The missing PCR fragment on Csdel 5DL-1 indicated the location on wheat long arm of chromosome 5D between the deletion segments 1 and 5.

Specificity of primers is the non-recurring binding in the target genome. This is reflected in a single PCR and a correct or syntenically localised amplicon. Fig 4 shows an example of the *Cbf1* amplicon localisation via NT- and deletion-lines.

The inspection of primer functionality and single PCR product generation is a standard for the development of primers and therefore is the first necessary step of the presented approach. Via the first inspection step we have eliminated 27.73% of studied primer pair combinations. Most of these showed no PCR amplification probably due to non-binding of target sequences. The second important step of checking the amplicon specificity is the mapping of the PCR products via NT-lines to get information about the correct amplification on the correct target chromosome template and sub-genome. By using NT-mapping of PCR amplicons we have eliminated 18.49% of primer pair combinations. One part of the eliminated PCR products shows a chromosome localisation that differs from what has been reported in the literature. In this case, we assume a non-specific binding in the wheat genome. That can occur if primers are derived from related organisms and not from wheat itself. For seven of eight discarded candidate genes, sequences of related organisms (*Triticum monococcum* and *Hordeum vulgare*) were used for primer development.The other part of eliminated primer pair combinations showed a PCR product on all NT-lines which may be due to the fact that both primers (forward and reverse) bind at least to two sub-genomes.

By using the draft wheat chromosome arm sorted sequences [10–12] and simple comparative methods we were able to develop gene specific primers in hexaploid wheat with a high success rate of 58.60%. Also a very high rate of 54.62% for specific fragment amplification confirmed the usefulness of wheat genomic sequence. To our knowledge such high rate is not yet described in literature for specific primer/marker development in polyploid plants. An overview of published success rates revealed a variation in microsatellite amplification in wheat between 22.88 and 45.0% [58–61]. In cotton this rate was 23.3% [62]. Contrary, Wang et al. [63] describe the development of effectively derived primers for sequence tagged sites (STS) with 24.56% and for STS primer combinations of only 3.7% in wheat. Chen et al. [64] achieved a rate of 27.5% for STS marker development in wheat. In *Brassica oleracea* (which is a paleohexaploid plant) a success rate of 29.1% is described in allele specific PCR primer development [65]. The highest success rate reported in literature is for potato [56]. In this study a rate of 51.79% developed intron targeting (IT) markers was achieved. With the ongoing genome sequencing projects and subsequent development of genome-wide physical maps in wheat and related plants an increase in the success of specific primer development may be expected.

### Sequencing of frost tolerance candidate genes and BLAST based verification

In this study 18 out of 19 (94.74%) frost tolerance genes were sequenced using the same primers used for PCR amplification. For gene *Cbf7*, for which initial sequencing failed, a set of newly designed sequencing primers improved the sequencing, therefore optimisation for single band products could be recommended as a part of the verification procedure. Concerning the gene *Tacr7*, Kocsy et al. [55] claimed BQ659345 of *Hordeum vulgare* is identical to the *Tacr7* gene in wheat. However, the analysis of the generated sequences presented in this paper showed an identity of 84% to the reference sequence L28093 for *Tacr7* of wheat and 92% to BQ659345. In contrast, our sequences reveal an identity of 92% to X97916 of *Hordeum vulgare* which is annotated as the barley low temperature gene 14.1 (*Blt14*.*1*). BLT14.1 shows a considerable homology to WLT10, as described by Ohno et al. [66]. Matching BQ659345 against X97916 results in an identity of 99%. Furthermore *Tacr7*, *Blt14*.*1* and *Wlt10* are located on chromosome 2 of barley and wheat, respectively [55, 66, 67]. We also mapped PCR fragments derived from *Tacr7* on chromosome 2B. Further BLAST results indicate that the sequence of our *Tacr7* is with 92% the initial sequence BQ659345. Furthermore, it was shown recently that the newest sequence of *Tacr7* [55] is very similar to the sequences of the genes *Blt14*.*1* and *Wlt10*, in contrast to the L28093 sequence (described also as *Tac7* [31]). The nucleotide identity of 99% between *Blt14*.*1* (X97916) and the initial reference sequence (BQ659345), which is published as *Tacr7* [55], backed this hypothesis. All other PCR fragment sequences have shown a very good sequence identity to the original gene of interest (97.5%).

The sequencing of single bands and correct chromosome assigned PCR amplicons followed by BLAST based verification is the last check-up step in the workflow presented in this study. The results of the BLAST based verification demonstrate that the selection of PCR single products and the assignment to the correct chromosomes of the PCR amplicons is an efficient instrument of locus specific primer selection. The combination of sequencing and BLAST based verification using the presented approach leads to very robust results with an error rate tending to zero.

The identified SNPs at 11 polymorphic candidate genes can be used for developing SNP based marker. Also the InDels in eight candidate genes are suited for marker development based on size polymorphisms. Based on these PCR amplicons can be employed for genetic mapping of correspondingcandidate genes in biparental mapping populations, thereby allowing for the first time their genetic localization.

This paper describes an efficient approach for the development of locus specific primers in wheat. With the aid of this locus specific primers are necessary for locus specific sequencing and detection of genes specific polymorphisms (SNPs and InDels) between genotypes of interest. The detected polymorphisms can follow up the use for genetic mapping, but also for gene editing via sequence information for transcription activator-like effector nucleases (TALENs) [68–70] or clustered regularly interspaced short palindromic repeats (CRISPR/Cas) systems [71]. Therefore our approach of development of locus specific primers is a base for many downstream applications i.e. detection of new polymorphisms, development of new markers, genetic mapping and gene editing in wheat.

## Conclusion

It is still difficult to develop molecular markers in *Triticum aestivum* due to the very complex genome. In this study we presented anefficient approach for gene and genome specific primer development by using sequence data of wheat. Altogether, we have developed specific primers for 19 out of 27 selected frost candidate genes. For 27 candidate genes 119 primer pairs were generated of which 65 were specific. Out of candidate gene specific primer fragments 36 fragments were selected, corresponding to 19 genes, for validation via sequencing. Finally, 35 amplicons could be successfully sequenced and only one specific sequence showed a low identity of approximately 83% to the original reference sequence.

By using the presented approach for gene specific primer/PCR development, it is possible to sequence and analyse interesting candidate genes in wheat by using gene information of related sequenced plant species. The wheat genome sequences currently available, in combination with the wheat physical map, are well suited for the development of specific primers. The approach for primer design, developed within this study turned out to be very efficient by using available wheat genomic resources and it is expected to perform even better once new versions of wheat genomic sequences will be available.

## Supporting Information

S1 Fig Chromosome localisation and cycler programs of the PCR fragments Cbf7, Ppd-B1f and Ppd-D1b via NT-lines. (TIF)

S1 Table Plant material of NT- and Deletion-lines. (XLSX)

S2 Table Candidate gene specific primers, primer specificity, PCR fragments, used polymerases, cycler programs and primers for fragment re-sequencing. Primer names with ^†^ are developed in course of this work but published from Keilwagen et al. [53]. Primer names with * as already published were used in combination with primers with ^†^ and without labels. ^1^[86]; ^2^[73]; ^3^[87]; ^4^[81]; ^5^[37]; ^6^ [53]. ^†^Primers published in Keilwagen et al. [53]. *Already published primers
(XLSX)

S3 Table Primer specificity and mismatches to compared the three sub-genomes of functional and correct localised PCR fragments via *in silico* alignments. Primers assigned ^†^ are developed in course of this study and published in Keilwagen et al. [54]. Already published primers with * assigned were used in combination with primers in green and black. The column differences describe the numbers of SNPs/InDels between primers at sequence level of A, B and D genomes. The columns position of InDels and SNPs in 5' to 3`direction describes the position of the differences between primers at sub-genomes (InDels and SNP) from primer 5´ to 3´ end direction. ^1^[86]; ^2^[73]; ^3^[87]; ^4^[81]; ^5^[37]; ^6^ [53]. ^†^Primers published in Keilwagen et al. [53]. *Already published primers.
(XLSX)
