# Supplementary figures and images for: An Efficient Approach for the Development of Locus Specific Primers in Bread Wheat (Triticum aestivum L.) and Its Application to Re-Sequencing of Genes Involved in Frost Tolerance
Source: PLoS One. 2015 Nov 13;10(11):e0142746. doi: 10.1371/journal.pone.0142746 (PMC4643983; doi:10.1371/journal.pone.0142746)

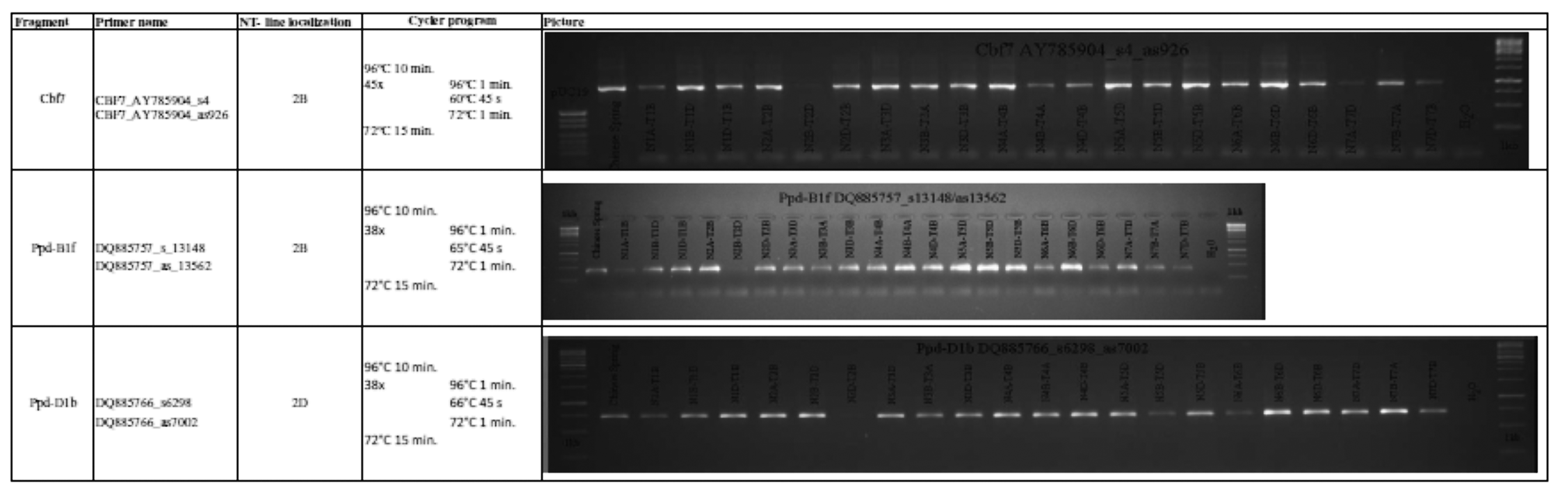

Supplement: S1 Fig — (TIF) [file pone.0142746.s001.tif]
